# KPMapNet: Keypoint Representation Learning for Online Vectorized High-Definition Map Construction

**DOI:** 10.3390/s25061897

**Published:** 2025-03-18

**Authors:** Bicheng Jin, Wenyu Hao, Wenzhao Qiu, Shanmin Pang

**Affiliations:** 1 School of Software Engineering, Xi’an Jiaotong University, Xi’an 710049, China; doubledig@stu.xjtu.edu.cn (B.J.); a2801551754@stu.xjtu.edu.cn (W.Q.); 2 China Academy of Electronics and Information Technology, Beijing 100041, China; haowenyu@stu.xjtu.edu.cn

**Keywords:** HD map construction, geometric modeling, autonomous driving, transformer

## Abstract

Vectorized high-definition (HD) map construction is a critical task in the autonomous driving domain. The existing methods typically represent vectorized map elements with a fixed number of points, establishing robust baselines for this task. However, the inherent shape priors introduce additional shape errors, which in turn lead to error accumulation in the downstream tasks. Moreover, the subtle and sparse nature of the annotations limits detection-based frameworks in accurately extracting the relevant features, often resulting in the loss of fine structural details in the predictions. To address these challenges, this work presents KPMapNet, an end-to-end framework that redefines the ground truth training representation of vectorized map elements to achieve precise topological representations. Specifically, the conventional equidistant sampling method is modified to better preserve the geometric features of the original instances while maintaining a fixed number of points. In addition, a map mask fusion module and an enhanced hybrid attention module are incorporated to mitigate the issues introduced by the new representation. Moreover, a novel point-line matching loss function is introduced to further refine the training process. Extensive experiments on the nuScenes and Argoverse2 datasets demonstrate that KPMapNet achieves state-of-the-art performance, with 75.1 mAP on nuScenes and 74.2 mAP on Argoverse2. The visualization results further corroborate the enhanced accuracy of the map generation outcomes.

## 1. Introduction

High-definition (HD) maps are critical components in autonomous driving systems, as they provide essential environmental information for tasks such as trajectory forecasting [1,2,3], path planning [4,5,6], and other downstream applications [7,8]. Traditionally, the creation of these maps has depended on the manual annotation of LiDAR point clouds, a process that is labor-intensive, time-consuming, and costly. Moreover, manual annotation procedures are not readily updated to reflect dynamic changes in road conditions. In response, recent studies [9,10,11,12,13] have explored leveraging onboard sensor data to generate HD maps for the surrounding environment in real time.

Several works [9,11,12,14,15] have formulated real-time HD map generation as a semantic segmentation problem, aiming to learn and produce pixel-level rasterized maps from a bird’s-eye view (BEV) perspective. However, rasterized maps inherently contain redundant pixel-level semantic information and are not directly compatible with downstream tasks. Consequently, additional post-processing is required to vectorize these maps, a procedure that not only increases computational overhead but also may introduces cumulative errors. To overcome these limitations, VectorMapNet [16] represents each map element as a sequence of points within a two-stage framework that progressively refines the predictions from coarse to fine levels. Subsequently, MapTR [13] employs DETR to directly regress the point coordinates of map elements before grouping the predicted points to form complete road element instances. MapTRv2 [17] further improves model accuracy and training efficiency by refining the attention mechanism and incorporating auxiliary supervision.

**Figure 1:**
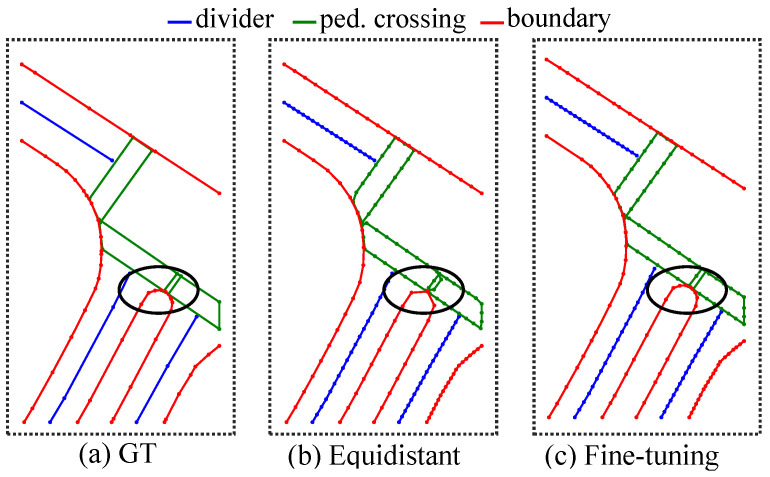
Different approaches to ground truth processing. (**a**) Ground truth provided by the dataset; (**b**) ground truth processed using equidistant sampling; (**c**) ground truth processed using keypoint fine-tuning. As highlighted in the circled region of (**b**), conventional ground truth processing methods often introduce additional shape distortions. In contrast, the proposed approach effectively preserves shape consistency, thereby mitigating these distortions.

Despite these promising advancements, the current map vectorization frameworks still exhibit several limitations. Representing map elements as point sets leads to varying numbers of points depending on their complexity, complicating model training. To address this variability, some studies [13,17] employ equidistant sampling on raw map data, converting each element into a fixed number of points regardless of shape complexity. However, as demonstrated in Figure 1b, this approach results in a loss of precision, particularly in regions characterized by right angles and curves, and introduces inherent errors that are not adequately captured by evaluation metrics, potentially impacting downstream tasks.

To overcome these issues and achieve a more precise map representation, the conventional equidistant sampling method has been refined by introducing a novel keypoint fine-tuning strategy. This approach selectively samples points to preserve the original shape of map elements as accurately as possible, thereby minimizing distortion. However, the resulting point sets no longer exhibit equidistant spacing, which introduces new challenges for network training. In response, KPMapNet is proposed as a novel framework designed to accurately model map elements through a redesigned loss function and an integrated prediction architecture that incorporates map masks. Specifically, the training ground truth is partitioned based on positional features into key points which delineate the shape and collinear points that supplement the representation. Based on this partitioning, the conventional point-to-point matching loss is reformulated into a point-to-line hybrid matching loss. Furthermore, a BEV mask fusion module is designed to enhance the localization and representational capabilities of BEV features by integrating the learned BEV mask into the feature map. Additionally, a hybrid query module is introduced within the decoder to improve query initialization and facilitate more effective detection.

Extensive experiments conducted on the NuScenes [18] and Argoverse2 [19] datasets demonstrate that KPMapNet achieves a state-of-the-art performance in terms of accuracy. Moreover, the visualization results indicate that KPMapNet yields more accurate shape recognition for map elements. The main contributions of this work can be summarized as follows:

- A keypoint fine-tuning strategy is proposed that optimizes the preservation of original ground truth shape features, establishing a novel method for ground truth representation in this field.
- A novel framework, KPMapNet, is introduced that integrates map mask feature fusion with an innovative point-to-line hybrid matching loss, thereby enabling the precise modeling of map elements.
- KPMapNet achieves 75.1 mAP on the NuScenes dataset and 74.2 mAP on the Argoverse2 dataset. Moreover, the visualization results demonstrate the framework’s improved predictive performance for instances exhibiting complex shapes.

## 2. Related Works

### 2.1. Online HD Map Construction

Traditional offline HD map generation typically relies on manual or semi-automated annotation [9,20]. With the emergence of methods that transition from picture view (PV) to BEV representations [21,22], recent studies [1,23,24,25] have investigated the feasibility of generating HD maps online directly using perspective view sensors. For example, HDMapNet [12] aims to directly generate vectorized map elements rather than rasterized maps, while VectorMapNet [16] employs a two-stage framework with an auto-regressive decoder to iteratively predict vector vertices. Furthermore, MapTR [13] and its enhanced version, MapTRv2 [17], introduce novel representations for map elements, enabling the simultaneous regression of both category and positional information. The proposed approach further extends these methods by incorporating generated map mask features into the model to better capture the specific vector shapes of map elements. In addition, KPMapNet introduces supplementary instance queries with shared decoder parameters during training to enhance overall model performance.

### 2.2. Map Instance Modeling

HD maps consist of diverse instances with varying geometric characteristics, such as lane dividers, pedestrian crossings, and road boundaries. In certain datasets [18], these map elements are typically stored and represented as polylines. Unlike the fixed-format bounding boxes used in detection tasks, the number of points in a polyline instance can vary, which poses significant challenges for network training. The conventional approaches [13,17,26] typically sample a fixed number of equidistant points from the ground truth, a strategy that may lead to a loss of shape information. In contrast, BeMapNet [24] represents ground truth using Bézier curves, while PivotNet [27] dynamically models and matches instances by employing pivotal points on a per-instance basis. In this paper, KpMapNet proposes a keypoint fine-tuning strategy that replaces equidistant sampling with a more precise representation that closely aligns with the original instances, thereby preserving detailed map features while maintaining model efficiency.

### 2.3. Map Mask for Segmentations

Image masks are widely employed in segmentation tasks to enhance the quality of both instance-level and semantic features. Previous studies [28] have examined the interaction between learned mask features and instance activation maps to effectively capture object-specific characteristics. Other works [29,30] have integrated mask features into transformer-based architectures, leveraging attention mechanisms for efficient feature extraction. In the context of HD map detection, KepMapNet utilizes high-quality map mask features to emphasize and enhance regions with detailed map annotations, thereby improving the model’s ability to capture fine-grained information.

## 3. The Proposed Method

### 3.1. Model Pipeline

**Figure 2:**
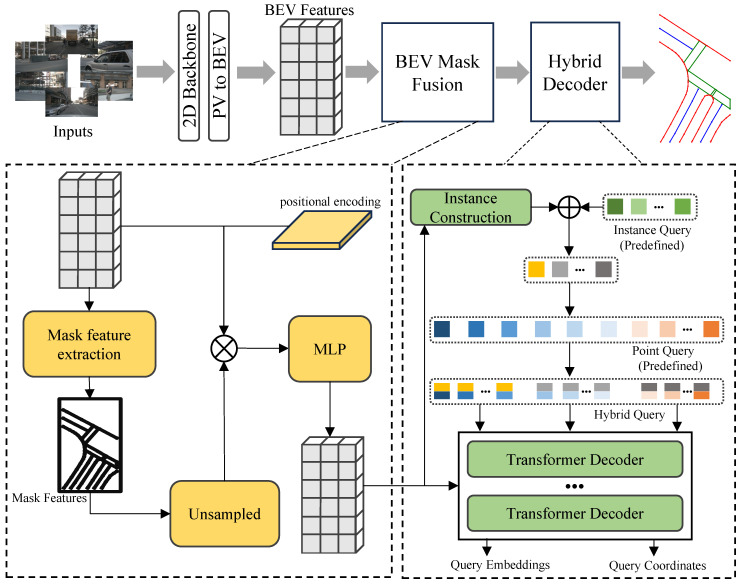
Overview of the KPMapNet framework. The top row illustrates the overall model pipeline, where multi-view images serve as input, and the model generates vectorized map elements in an end-to-end manner. The BEV mask fusion module extracts map masks and then refines BEV features through a reverse fusion process, enhancing spatial and semantic consistency. The hybrid decoder dynamically interacts to extract both point-level and element-level information from the map elements, continuously constructing and updating queries for improved representation learning.

Figure 2 illustrates the overall architecture of KPMapNet. Building upon MapTRv2 [17], KPMapNet first extracts features from the input of surrounding PV images and projects them into the BEV space. To enhance the representational capacity of the BEV features, a BEV mask fusion module is introduced, which reintegrates the generated rasterized map into the BEV features. These enriched BEV features are subsequently fed into a carefully designed hybrid attention decoder that generates the corresponding map instances. Moreover, this method has been redesigned to optimize the processing of the ground truth data to obtain more accurate training targets; the loss function has also been redefined accordingly.

The remainder of this section is organized as follows: Section 3.2 details the proposed keypoint fine-tuning sampling method, Section 3.3 describes the architecture of KPMapNet, and Section 3.4 introduces the overall training loss function.

### 3.2. Ground Truth Representation

Each map instance is defined by its class label c and an ordered sequence of points $P={\left\{x_{i},y_{i}\right\}}_{i=1}^{N}$ that describe its shape, where *N* specifies the number of points representing each instance. The connectivity between the points is implicitly encoded in their ordering. As illustrated in Figure 1, the number of points *N* in the original representation varies with the complexity of the map element, complicating the training process. To standardize this representation, equidistant sampling is applied, resulting in a fixed number of points with equal spacing. While this approach simplifies the learning task, it can introduce geometric errors.

To mitigate this issue, this research decomposes the original point set P into the following two subsets: a set of key points $P_{key}$, which capture the essential shape features, and a set of redundant collinear points $P_{col}$, which serve as fillers. The keypoint set $P_{key}$ is an ordered series of points that delineate the overall shape, typically marking changes in direction, whereas $P_{col}$ does not contribute additional shape information.

Let $P_{equ}$ denote the point set obtained via equidistant sampling. Then, adjust $P_{equ}$ using $P_{key}$ to preserve the original shape. The keypoint fine-tuning procedure is as follows: First, simplify P using methods such as Douglas–Peucker [31] or Visvalingam–Whyatt [32] to derive $P_{key}$. Then, apply equidistant sampling to P to obtain $P_{equ}$. A custom matching algorithm is designed to align these two sets and obtain a fine-tuned configuration.

Consider matching the keypoint sequence $P_{key}={\left\{x_{i},y_{i}\right\}}_{i=1}^{K}$ with the equidistant sequence $P_{equ}={\left\{x_{i},y_{i}\right\}}_{i=1}^{M}$, where *K* is the number of key points and *M* is a predefined fixed number. While *M* is fixed, *K* varies with the map element’s shape complexity. Let ν denote the set of all possible combinations given the ordering constraints; there are $C_{M-2}^{K-2}$ possible combinations. For the *i*-th key point $\left(x_{i},y_{i}\right)$, let $\left(x_{\nu (i)},y_{\nu (i)}\right)$ be its corresponding point in $P_{equ}$. For a given matching combination ν, the matching cost is defined as follows:

(1) $$L_{match}\left(P_{equ},P_{key},\nu \right)=\sum_{i=1}^{K}∥\left(x_{i},y_{i}\right),\left(x_{\nu (i)},y_{\nu (i)}\right)∥,$$

where $∥\cdot ∥$ denotes $L_{2}$ distance. The optimal matching $\nu_{b}$ is then obtained by minimizing the matching cost, as in the following equation:

(2) $$\nu_{b}=\underset{\nu^{*}\in \nu }{\mathrm{argmin}}L_{match}\left(P_{equ},P_{key},\nu^{*}\right)$$

Due to the ordered nature of the point sets, a fixed correspondence is assumed, i.e., ν(1)=1 and ν(K)=M. This matching problem is addressed using dynamic programming. The algorithm uses an array dp to store the matching costs; let dp[i][j] denote the minimum matching cost for the first *i* key points and the first *j* equidistant points. The state transition can be expressed as follows:

(3) $$dp\left[i\right]\left[j\right]=\min(dp\left[i\right]\left[j-1\right],dp\left[i-1\right]\left[j-1\right]+∥\left(x_{i},y_{i}\right),\left(x_{j},y_{j}\right)∥)$$

The initial condition is defined as follows:

(4) $$dp\left[1\right]\left[j\right]=\min_{1<=k<=j}∥\left(x_{1},y_{1}\right),\left(x_{k},y_{k}\right)∥,1<=j<=M-K$$

Finally, replacing $P_{equ}$ with the optimal combination obtained $\nu_{b}$ yields the fine-tuned keypoint set $P_{fin}={\left\{x_{i},y_{i}\right\}}_{i=1}^{M}$, which maintains a consistent number of points while preserving a more accurate representation of the original shapes.

### 3.3. Architecture Detail

**BEV Feature Extractor.** Given the input surround images $I=\left\{I_{1},\ldots ,I_{n}\right\}$, KpMapNet employs the conventional CNN backbones [33,34,35], along with an FPN [36], to extract the multi-view PV features $F=\left\{F_{1},\ldots ,F_{n}\right\}$. These PV features are then integrated into a unified BEV representation using various strategies such as LSS [23,25,37,38,39], GKT [40], CVT [11] and Deformable Attention [13,41,42]. Following the settings in MapTRv2 [17], KpMapNet adopts LSS [38] as the default transformation method to leverage depth information during supervised learning. The resulting BEV feature is denoted as $F_{bev}\in R^{H\times W\times C}$, where H×W represents the spatial dimensions and *C* denotes the feature depth.

**BEV Mask Fusion.** Before passing the BEV features to the decoder, KpMapNet introduces an optimization module that refines the BEV features at a fine-grained, point-level resolution by leveraging a rasterized BEV feature mask. As shown in Figure 2, a convolutional operation is applied to $F_{bev}$ to generate the BEV mask $F_{mask}\in R^{1\times H\times W}$. During training, $F_{mask}$ is supervised using the ground truth mask ${\mathrm{GT}}_{mask}$, with the loss function defined as the cross-entropy loss, as in the following equation:

(5) $$L_{mask}=L_{CE}\left(F_{mask},{\mathrm{GT}}_{mask}\right).$$

Subsequently, a CNN $\phi_{up}\left(\cdot \right)$ upsamples $F_{mask}$ to a channel dimensionality of 32. This upsampled mask is concatenated with the BEV feature $F_{bev}$ and a two-dimensional normalized positional encoding $F_{pose}\in R^{2\times H\times W}$ that encodes spatial location. Finally, these features are fused via a convolutional operation, as follows:

(6) $$F_{fus}=\mathrm{Conv}\left(\mathrm{Concat}\left(F_{bev},\phi_{up}\left(F_{mask}\right),F_{pose}\right)\right).$$

The fused feature $F_{fus}\in R^{H\times W\times C}$ emphasizes the salient positional and semantic information, thereby enhancing the model’s ability to accurately predict detailed map shapes by effectively distinguishing instance features from background noise.

**Hybrid Attention Decoder.** For each map instance, the transformer decoder employs a set of queries to predict both the category and the geometric details through regression. As depicted in Figure 2, KpMapNet adopts a hybrid approach that combines instance queries $Q_{ins}$ with point queries $Q_{pts}$, while leveraging BEV features to refine the instance queries for improved initialization. The instance queries $Q_{ins}\in R^{D\times C}$, with *D* denoting the number of instance queries, are dynamically updated alongside their random initialization to incorporate instance-specific features. Specifically, a convolutional layer followed by a sigmoid activation predicts an instance mask, which is then fused with the randomly initialized instance queries, as expressed in the following equation:

(7) $$Q_{ins}=\mathrm{Sigmoid}\left(\mathrm{Conv}\left(F_{fus}\right)\right)\times F_{fus}^{⊺}+\mathrm{Init}\left(Q_{ins}\right).$$

The point queries $Q_{pts}\in R^{M\times C}$ are obtained via random initialization. These instance and point queries are then combined to form the hybrid queries $Q\in R^{D\times M\times C}$. Finally, Q and $F_{fus}$ are fed into an *L*-layer decoder to generate the predicted category $\hat{c}$ and the point set $\hat{P}={\left\{{\hat{x}}_{i},{\hat{y}}_{i}\right\}}_{i=1}^{M}$. The iterative residual design of the decoder further enhances the network’s capacity to learn robust regression features, thereby improving both representation and prediction accuracy.

### 3.4. Training Loss

**Figure 3:**
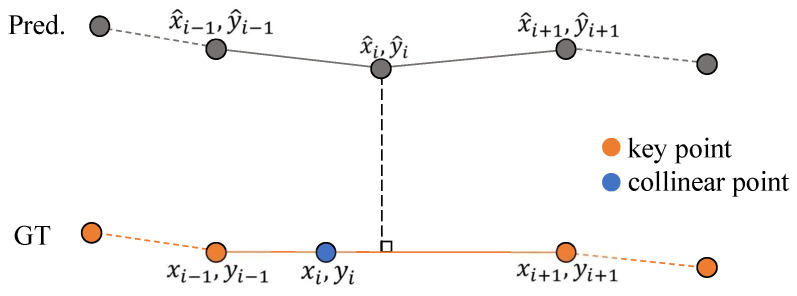
Schematic of point-to-line matching. For clarity, the gap between the predicted points and the ground truth is exaggerated.

**Point–line Matching Loss.** The fine-tuned point set $P_{fin}$ obtained via the keypoint fine-tuning strategy does not enforce equal spacing between points, which poses new challenges for training. To accommodate the characteristics of this new ground truth, the original point-to-point matching loss is modified. Specifically $P_{fin}$ can be decomposed into key points $P_{key}$ and collinear filler points $P_{col}$. For points corresponding to key features, the L1 distance is computed directly. For collinear points, the L1 distance is calculated relative to the perpendicular projection onto the predicted line, as illustrated in Figure 3. The final point-to-line matching loss is defined as follows:

(8) $$L_{p2l}=L_{1}\left(P_{key},{\hat{P}}_{key}\right)+L_{1}\left(\mathrm{Line}\left(P_{col}\right),{\hat{P}}_{col}\right),$$

where Line(·) denotes the straight line on which the collinear points reside.

**Overall Loss.** Following the approach in MapTRv2 [17], KpMapNet also incorporates classification loss $L_{cls}$ and an edge direction loss $L_{dir}$. The total loss function is defined as follows:

(9) $$L=\beta_{1}L_{mask}+\beta_{2}L_{p2l}+\beta_{3}L_{cls}+\beta_{4}L_{dir},$$

where $\beta_{1}$, $\beta_{2}$, $\beta_{3}$ and $\beta_{4}$ are the corresponding loss weights.

## 4. Experiments

### 4.1. Datasets

KpMapNet evaluates model performance through comprehensive experiments on two widely recognized datasets in autonomous driving research, namely NuScenes [18] and Argoverse2 [19]. The NuScenes dataset comprises 1000 driving scenarios collected in Singapore and Boston, USA, under diverse weather conditions and times of day. Out of these, 750 scenes are allocated for training, 100 for validation, and 150 for testing. Each scenario spans approximately 20 s and provides 40 keyframes sampled at 2 Hz, including 360-degree RGB images from six cameras, LiDAR point clouds, and precisely annotated 2D map instances. Argoverse2 consists of 1000 scenarios with 3D map annotations collected from six U.S. cities, featuring data from LiDAR, stereo cameras, and ring cameras. In line with the previous studies [16,17,26], our experiments focus on the following three static categories of map elements: pedestrian crossing (ped.), lane divider (div.), and road boundary (bou.).

### 4.2. Evaluation Metrics

To ensure fair comparisons, the quality of map instance predictions is evaluated using average precision (AP) based on the average Chamfer distance. The Chamfer distance quantifies the alignment between predictions and ground truth by computing the mean Euclidean distance between corresponding points. The method first samples points from the predicted instances and computes the forward Chamfer distance as the mean of the shortest distances from predictions to the ground truth, then computes the reverse distance in the same manner for the ground truth. The average Chamfer distance is obtained by averaging these two measures. Predictions with distances below a specified threshold are considered true positives (TPs). Following the previous studies [17,27], this research employs two threshold sets, {0.5,1.0,1.5} m and {0.2,0.5,1.0} m, and reports the mAP as the average precision across these thresholds. Consistent with MapTRv2 [17], 100 points are sampled for Chamfer distance calculations.

### 4.3. Implementation Details

**Architecture.** KPMapNet employs ResNet50 [33], EfficientNet-B0 [34], and VoVNetV2-99 [35] as its backbone networks. For the NuScenes dataset, the original image resolution is 1600×900. These images are resized by a factor of 0.5 and padded to 800×480. In the Argoverse2 dataset, the seven camera images have differing resolutions (with the front view at 1550×2048 and the others at 2048×1550). The front view images are cropped to 1550×1550 and padded to match the other views (2048×1550), and then all seven images are resized to 704×544. The preprocessed images are subsequently passed through the backbone and FPN for feature extraction, followed by an encoder to generate BEV features. In the experiments, the BEV perception range is set to 30 m both ahead and behind the vehicle, as well as 15 m to both the left and right. With a feature resolution of 0.15×0.15 m, this results in a grid of 200×100 BEV queries, each with a feature dimension of 256, processed by six decoder layers. The default number of instance queries is 50, and the number of point queries is 20. All settings are adopted from the previous studies [13,17,27] to ensure a fair comparison.

**Training and Inference.** KpMapNet is trained on a single NVIDIA A100 Tensor Core GPU (Santa Clara, CA, USA) with a batch size of 16, utilizing the AdamW [43] optimizer with a weight decay of 0.01. Training is conducted for 24 epochs by default, starting with an initial learning rate of $4\times 10^{-4}$ and applying a decay factor of 0.1 for the backbone. The learning rate follows a cosine annealing schedule with a linear warm-up phase. For the point-line matching loss, the loss weight is set to 6, while all other hyperparameters adhere to the configurations used in MapTRv2 [17]. Inference is executed on a single NVIDIA GeForce RTX 3090 GPU, with the inference time duly recorded.

### 4.4. Main Results

**Figure 4:**
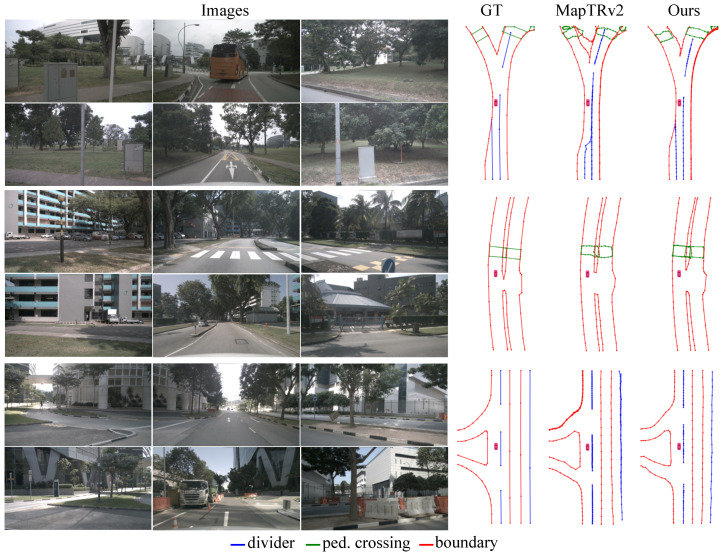
Comparison of visualization results on the NuScenes dataset.

**Results on nuScenes.** KPMapNet is trained using various epoch schedules and backbone networks on the nuScenes dataset. Table 1 presents a performance comparison of KPMapNet, using only RGB image inputs, alongside the previous methods. KPMapNet achieves better performance (75.1, 53.2 mAP) under both threshold configurations. The reproducibility metrics for the comparison methods [12,13,16,17,27,44] are obtained by executing publicly available source code and model checkpoints. The results demonstrate that KPMapNet outperforms the previous approaches, yielding significant improvements in complex features, particularly for ped. and bou. Under the easy threshold {0.5,1.0,1.5} m, KpMapNet-ResNet50 shows improvements of 3.9 mAP for ped. and 3.7 mAP for bou., compared to the previous SOTA MapTRv2. Additionally, KPMapNet outperforms PivotNet by 3.6 mAP for ped. and 3.7 mAP for bou. KpMapNet based on VoVNetV2-99 achieves 75.1 mAP—1.7 mAP higher than MapTRv2. Moreover, the visualizations in Figure 4 illustrate the improved shape representation achieved by our model.

**Results on Argoverse2.** As shown in Table 2, on the Argoverse2 dataset, KPMapNet consistently exceeds teh previous SOTA methods under both evaluation settings. Our method achieves competitive performance, with KpMapNet outperforming MapTRv2 by reaching 74.2 mAP, thereby further validating the effectiveness of our proposed approach.

### 4.5. Ablation Study

This section presents the ablation experiments used to evaluate the contributions of the proposed modules and design choices. Unless otherwise specified, the experiments were conducted using ResNet50 [33] as the backbone, with NuScenes camera images as input, and training was performed for 24 epochs under the easy threshold configuration.

**Ablation on different modules.** The ablation results in Table 3 validate the contribution of each design component to overall performance. Initially, the configurations of MapTRv2 were adopted as baselines for comparison. Although the introduction of keypoint fine-tuning initially increased the network’s learning complexity and caused a slight performance decline, the subsequent integration of additional components resolved this issue and enhanced overall performance. Specifically, the new ground truth representation significantly improved the network’s precision in detecting complex vectors. The point-line matching loss accelerated convergence while maintaining performance comparable to the baseline. Moreover, incorporating mask fusion and hybrid attention resulted in performance gains of 2.9 mAP and 1.5 mAP, respectively. Together, these components achieved a cumulative improvement of 3.8 mAP, which was the highest observed enhancement.

**Point–line loss weight.** This research explores various weight configurations for the point-line matching loss to determine the optimal setting. As shown in Table 4, performance was relatively insensitive to specific weight values; however, excessively high or low weights adversely affected network convergence, leading to performance degradation. Based on these observations, a weight of 6 was identified as the optimal choice.

**Auxiliary mask loss.** Table 5 demonstrates the effectiveness of BEV mask supervision. By enforcing constraints on BEV features to ensure they capture meaningful spatial information, the auxiliary mask loss $L_{mask}$ yielded a 1.7 mAP improvement, thereby enhancing overall performance.

**Discussion of different thresholds.** Smaller threshold settings impose stricter performance requirements on the model. Given that HD maps for autonomous driving require error control at the centimeter level, performance improvements under these stricter thresholds are particularly valuable for practical applications. As shown in Table 6, this model consistently achieves superior results compared to the previous methods across all thresholds. The improvements are especially pronounced at the 0.2 m and 0.5 m thresholds, underscoring the model’s effectiveness under more rigorous evaluation criteria.

## 5. Conclusions

This paper addresses the challenges of accurately processing vector representations for map elements by introducing a novel keypoint fine-tuning strategy and an online HD vectorized map detection framework, KPMapNet. Our approach offers a unified method for refining map elements, effectively preserving intricate shape details that are typically compromised during traditional ground truth processing. By integrating innovative components—including a BEV mask fusion module, a hybrid attention mechanism, and a point-line matching loss function—KPMapNet mitigates the loss of fine structural information and enhances the precision of HD map construction. Experimental results on public datasets demonstrate that KPMapNet achieves state-of-the-art performance, establishing a new benchmark in the domain. Notably, our framework significantly improves the detection accuracy of complex map elements, such as pedestrian crossings and road boundaries, underscoring its potential for practical autonomous driving applications.

Future research will explore the integration of multimodal sensor data and temporal information to construct more holistic and robust representations of the driving environment. These advancements are expected to further enhance the performance of vectorized map detection systems and contribute to the development of more reliable autonomous driving solutions. 

## Figures and Tables

**Table 1:** Performance comparison on NuScenes dataset.

Method	Backbone	Epoch	AP_div._	AP_ped._	AP_bou._	mAP	AP_div._	AP_ped._	AP_bou._	mAP	FPS
			{0.5,1.0,1.5}m				{0.2,0.5,1.0}m				
HDMapNet [12]	EB0	30	23.6	24.1	43.5	31.4	17.7	13.6	32.7	21.3	0.8
KpMapNet (Ours)	EB0	30	26.2	33.5	45.3	35.0	14.5	15.8	25.0	18.4	14.1
VectorMapNet [16]	R50	110	47.3	36.1	39.3	40.9	27.2	18.2	18.4	21.3	2.2
MapTR [13]	R50	24	51.5	46.3	53.1	50.3	30.7	23.2	28.2	27.3	**15.1**
MapVR [44]	R50	24	54.4	47.7	51.4	51.2	-	-	-	-	**15.1**
PivotNet [27]	R50	24	56.5	56.2	60.1	57.6	41.4	34.3	39.8	38.5	10.4
MapTRv2 [17]	R50	24	62.4	59.8	62.4	61.5	40.0	35.4	36.3	37.2	14.1
MapTRv2 ^†^ [17]	R50	24	56.0 ^†^	57.3 ^†^	62.0 ^†^	58.4 ^†^	37.2 ^†^	31.9 ^†^	37.0 ^†^	35.4 ^†^	14.1
KpMapNet (Ours)	R50	24	62.4	63.7	66.1	64.1	42.7	37.9	43.5	41.4	13.9
MapTRv2 [17]	V2-99	24	67.1	63.6	69.2	66.6	-	-	-	-	9.9
MapTRv2 [17]	V2-99	110	73.7	71.4	75.0	73.4	-	-	-	-	9.9
KpMapNet (Ours)	V2-99	24	65.9	63.7	71.2	66.9	45.9	37.2	48.5	43.9	9.3
KpMapNet (Ours)	V2-99	110	**74.2**	**73.4**	**77.6**	**75.1**	**56.3**	**47.1**	**56.1**	**53.2**	9.3

^†^ indicates the results obtained by retraining with the keypoint fine-tuning GT. “-” means that the corresponding results are not available. “EB0”, “R50”, and “V2-99”, respectively, correspond to EfficientNet-B0 [34], ResNet50 [33], and VoVNetV2-99 [35]. Best results are highlighted in bold.

**Table 2:** Performance comparison on Argoverse2 dataset.

Method	Backbone	Epoch	AP_div._	AP_ped._	AP_bou._	mAP	AP_div._	AP_ped._	AP_bou._	mAP
			{0.5,1.0,1.5}m				{0.2,0.5,1.0}m			
VectorMapNet [16]	R50	-	36.1	38.3	39.2	37.9	-	-	-	-
MapTR [13]	R50	6	58.1	54.7	56.7	56.5	42.2	28.3	33.7	34.8
MapVR [44]	R50	-	60.0	54.6	58.0	57.5	-	-	-	-
PivotNet [27]	R50	6	-	-	-	-	47.5	31.3	43.4	40.7
MapTRv2 [17]	R50	6	**72.1**	62.9	67.1	67.4	52.5	34.8	40.6	42.6
KpMapNet (Ours)	R50	6	69.4	**74.7**	**78.5**	**74.2**	**53.3**	**45.7**	**56.1**	**51.7**

“-” means that the corresponding results are not available. Best results are highlighted in bold.

**Table 3:** Effectiveness of different modules in KPMapNet.

Fine-Tuning	Point-Line	Fusion	Hybrid	AP_div._	AP_ped._	AP_bou._	mAP
✗	✗	✗	✗	61.2	58.9	61.5	60.5
✓	✗	✗	✗	55.7	57.5	61.5	58.2
✓	✓	✗	✗	57.6	59.9	63.3	60.3
✓	✓	✓	✗	61.2	63.1	65.2	63.2
✓	✓	✗	✓	59.3	60.5	64.5	61.8
✓	✓	✓	✓	**62.4**	**63.7**	**66.1**	**64.1**

Best results are highlighted in bold.

**Table 4:** Effectiveness of point-line loss weight.

Loss Weight	AP_div._	AP_ped._	AP_bou._	mAP
4	61.0	63.0	66.6	63.5
5	61.3	64.5	65.9	63.9
6	**62.4**	**63.7**	**66.1**	**64.1**
7	61.3	61.6	65.7	62.9

Best results are highlighted in bold.

**Table 5:** Effectiveness of auxiliary loss.

$L_{\mathrm{mask}}$	AP_div._	AP_ped._	AP_bou._	mAP
✗	59.3	63.0	64.9	62.4
✓	**62.4**	**63.7**	**66.1**	**64.1**

Best results are highlighted in bold.

**Table 6:** Comparison of performance under different thresholds.

Method	mAP_0.2m_	mAP_0.5m_	mAP_1.0m_	mAP_1.5m_
VectorMapNet [16]	1.1	16.6	46.2	64.0
MapTR [13]	2.2	24.7	55.1	70.1
MapTRv2 [17]	5.7	38.6	67.3	72.9
KpMapNet (Ours)	**11.3**	**43.4**	**69.5**	**79.4**

Best results are highlighted in bold.

## Data Availability

The original contributions presented in this study are included in the article. Further inquiries can be directed to the corresponding author.
